# Risk of Subjection to Violence and Perpetration of Violence in Persons With Psychiatric Disorders in Sweden

**DOI:** 10.1001/jamapsychiatry.2019.4275

**Published:** 2020-01-15

**Authors:** Amir Sariaslan, Louise Arseneault, Henrik Larsson, Paul Lichtenstein, Seena Fazel

**Affiliations:** 1 Warneford Hospital, Department of Psychiatry, University of Oxford, Oxford, United Kingdom; 2 Social and Public Policy Unit, Faculty of Social Sciences, University of Helsinki, Helsinki, Finland; 3 Social, Genetic, and Developmental Psychiatry Centre, Institute of Psychiatry, Psychology, and Neuroscience, King’s College London, London, United Kingdom; 4 Department of Medical Epidemiology and Biostatistics, Karolinska Institutet, Stockholm, Sweden; 5 Orebro University School of Medical Sciences, Orebro, Sweden

## Abstract

**Importance:**

Key outcomes for persons with psychiatric disorders include subjection to violence and perpetration of violence. The occurrence of these outcomes and their associations with psychiatric disorders need to be clarified.

**Objective:**

To estimate the associations of a wide range of psychiatric disorders with the risks of subjection to violence and perpetration of violence.

**Design, Setting, and Participants:**

A total of 250 419 individuals born between January 1, 1973, and December 31, 1993, were identified to have psychiatric disorders using Swedish nationwide registers. Premorbid subjection to violence was measured since birth. The patients were matched by age and sex to individuals in the general population (n = 2 504 190) and to their full biological siblings without psychiatric disorders (n = 194 788). The start date for the patients and control groups was defined as the discharge date of the first psychiatric episode. The participants were censored either when they migrated, died, experienced the outcome of interest, or reached the end of the study period on December 31, 2013. Data were analyzed from January 15 to September 14, 2019.

**Exposures:**

Patients with common psychiatric disorders (eg, schizophrenia, bipolar disorder, depression, and anxiety) were differentiated using a hierarchical approach. Patients with personality disorders and substance use disorders were also included.

**Main Outcomes and Measures:**

Subjection to violence was defined as an outpatient visit (excluding a primary care visit), inpatient episode, or death associated with any diagnosis of an injury that was purposefully inflicted by other persons. Perpetration of violence was defined as a violent crime conviction. Stratified Cox regression models were fitted to account for the time at risk, a range of sociodemographic factors, a history of violence, and unmeasured familial confounders (via sibling comparisons).

**Results:**

Among 250 419 patients (55.4% women), the median (interquartile range) age at first diagnosis ranged from 20.0 (17.4-24.0) years for alcohol use disorder to 23.7 (19.9-28.8) years for anxiety disorder. Compared with 2 504 190 matched individuals without psychiatric disorders from the general population, patients with psychiatric disorders were more likely to be subjected to violence (7.1 [95% CI, 6.9-7.2] vs 1.0 [95% CI, 0.9-1.0] per 1000 person-years) and to perpetrate violence (7.5 [95% CI, 7.4-7.6] vs 0.7 [95% CI, 0.7-0.7] per 1000 person-years). In the fully adjusted models, patients with psychiatric disorders were 3 to 4 times more likely than their siblings without psychiatric disorders to be either subjected to violence (adjusted hazard ratio [aHR], 3.4 [95% CI, 3.2-3.6]) or to perpetrate violence (aHR, 4.2 [95% CI, 3.9-4.4]). Diagnosis with any of the specific disorders was associated with higher rates of violent outcomes, with the sole exception of schizophrenia, which was not associated with the risk of subjection to violence.

**Conclusions and Relevance:**

In this study, persons with psychiatric disorders were 3 to 4 times more likely than their siblings without psychiatric disorders to have been subjected to violence or to have perpetrated violence after the onset of their conditions. The risks of both outcomes varied by specific psychiatric diagnosis, history of violence, and familial risks. Clinical interventions may benefit from targeted approaches for the assessment and management of risk of violence in people with psychiatric disorders.

## Key Points

**Question:**

What is the incidence of subjection to violence or perpetration of violence in persons with psychiatric disorders?

**Findings:**

In this nationwide cohort study of 250 419 individuals with psychiatric disorders in Sweden, in the decade after the onset of their conditions, fewer than 7% of patients had either been subjected to violence severe enough to require specialist medical treatment or had perpetrated violence.

**Meaning:**

Persons with psychiatric disorders were approximately 3 to 4 times more likely than their siblings without psychiatric disorders to be either subjected to violence or to perpetrate violence.

## Teaser

This nationwide Swedish cohort study examines the risks of subjection to violence and perpetration of violence among patients with psychiatric disorders compared with siblings and individuals from the general population without psychiatric disorders.

## Introduction

Individuals diagnosed with psychiatric disorders may experience a range of adverse outcomes, with elevated risks of premature mortality,^1^ suicide,^1^ unemployment,^2^ and homelessness.^3^ In addition, these individuals have more contact with the criminal justice system and an increased risk of engaging in violent crime compared with the general population and with their siblings without psychiatric disorders.^4,5,6^ The evidence base regarding perpetration risks needs to be interpreted in the context of subjection to violence among individuals with psychiatric disorders. Reviews of previous research, expert opinion, and advocacy groups report that the rate of subjection to violence is considerably higher than the rate of perpetration of violence, and it is commonly suggested that this rate is elevated approximately 10-fold.^7,8^

However, evidence for increased rates of subjection to violence in individuals with psychiatric disorders is limited. First, systematic reviews have reported large but imprecise relative risks, ranging from a factor of 2 to 140, for subjection to violence in individuals with any psychiatric disorder compared with the general population.^9,10,11,12^ These reviews have primarily been based on cross-sectional studies using small and selected clinical samples that have relied on retrospective self-reports.^13,14^ Some of these limitations have been addressed by 2 population-based studies.^15,16^ However, these investigations did not adequately control for premorbid subjection to violence; hence, they were unable to exclude the possibility of reverse causation (ie, subjection to violence as the cause of the psychiatric disorder rather than vice versa) given the evidence indicating an association between subjection to violence in early life (and its related trauma) and psychiatric morbidity in adulthood.^17,18^

Second, twin and family studies have reported that psychiatric disorders, violent crime, and subjection to violence tend to aggregate in families, but their etiological associations remain poorly understood.^5,19,20^ The literature suggests, however, that the estimates in the studies examining associations between psychiatric disorders and subjection to violence may have been biased upwards because of substantial unmeasured familial confounding.

Third, only a few studies have explicitly considered the co-occurrence of subjection to violence and perpetration of violence. This point is notable because previous studies have suggested that the co-occurrence may represent a distinct subgroup that is differentially associated with psychiatric disorders.^20,21^

To address these gaps in knowledge, we conducted a study of the entire Swedish population born between January 1, 1973, and December 31, 1993. This approach allowed us to assess the potential associations of a wide range of psychiatric disorders with the risks of subjection to and perpetration of violence while accounting for unmeasured familial confounding.

## Methods

### Data Collection

All Swedish residents are assigned a unique 10-digit civic registration number, which is used in different nationwide registers and provides accurate linkage.^22^ We received deidentified data from Statistics Sweden after the study was approved by the regional research ethics committee of Karolinska Institutet. Informed consent is not a requirement for nationwide register-based studies in Sweden.^23^

The Multi-Generation Register provided data on all individuals born in Sweden and their biological parents, which enabled identification of full biological siblings. The National Patient Register provided data on all inpatient hospitalization episodes (*International Classification of Diseases, Revision 8* [*ICD-8*], *International Classification of Diseases, Ninth Revision [ICD-9]*, and *International Statistical Classification of Diseases and Related Health Problems, Tenth Revision [ICD-10]* data from 1973-2013) and specialist outpatient care visits (*ICD-10* data from 2001-2013) and is comprehensive of Swedish universal health care coverage. Violent crime convictions were derived from the National Crime Register, which includes information on criminal convictions beginning in 1973. Data on sociodemographic factors were gathered from census registers. The Migration Register and the Causes of Death Register, respectively, provided emigration and mortality dates. This study followed Strengthening the Reporting of Observational Studies in Epidemiology (STROBE) guidelines for cohort studies (eTable 1 in the Supplement).

Subjection to violence was defined as an outpatient visit (excluding primary care), inpatient care episode, or death related to any diagnosis of an injury purposefully inflicted by other persons (*ICD* codes and validation information in eMethods and eTable 2 in the Supplement). Violent perpetration was defined as a conviction for homicide, assault, robbery, violence against an officer, arson, or sexual offenses (excluding prostitution, solicitation of prostitution, or possession of child pornography). Individuals are convicted in Swedish courts irrespective of psychiatric disorder, although sentencing may be informed by such conditions. The patient data were not used to determine perpetration of violence status, and the conviction data were not used to determine subjection to violence status.

From a population sample of all individuals born in Sweden between January 1, 1973, and December 31, 1993 (eMethods in the Supplement), we identified all patients diagnosed with a psychiatric disorder older than 15 years (n = 250 419). Premorbid subjection to violence was measured since birth. We adopted a hierarchical approach to differentiate between schizophrenia, bipolar disorder, depression, and anxiety disorder (*ICD* codes in eTable 2 in the Supplement). We also examined patients with personality disorders, alcohol use disorders, and drug use disorders.

### Control Groups

We individually matched each patient by sex and birth year with 10 individuals in the general population who did not have that particular psychiatric disorder. Participants in the general population control group had to be alive and Swedish residents at the date of matching (eg, when the index person first received the psychiatric diagnosis). Patients could be matched with multiple individuals because the analyses only considered the associations within each cluster of patients and individuals from the general population. We also matched the patients with their full biological siblings who did not have psychiatric disorders to assess the role of unmeasured familial confounding. To maintain a high degree of statistical power, we analyzed all potential sibling pairs in the main analyses, with covariate adjustments for age and sex. The sibling comparison approach allowed us to account for all time-constant unmeasured familial confounding factors shared between siblings (eg, half of their cosegregating genes and their shared childhood environments). The extent to which the sibling comparisons were attenuated compared with the population estimates indicated the influence of unmeasured familial confounding.

The start date for the patients and control groups was defined as the discharge date of the first psychiatric episode. The participants were censored either when they migrated, died, experienced the outcome of interest, or reached the end of the study period on December 31, 2013.

### Statistical Analysis

We quantified the associations between psychiatric disorders and subjection to and perpetration of violence by fitting stratified Cox models that estimated adjusted hazard ratios (aHRs). Because each person diagnosed with a psychiatric disorder and their matches in the general population and sibling control groups were separately defined as unique strata, the model used this information to estimate varying baseline hazard rates across each combination of patients and individuals in the control groups. This approach implies that the comparisons were made within each stratum.

We initially fitted a crude model that accounted for sex and birth year (model 1). We subsequently further adjusted the model for birth order and parental background factors (model 2) as well as the individual’s history of subjection to violence and perpetration of violence (model 3). In model 4, we adjusted for unmeasured familial risks by refitting model 3 on the subsamples of differentially affected siblings. Model 4 was then refitted to each sex separately to assess moderation effects by sex. We examined the associations between specific psychiatric disorders and outcomes by testing each of them individually (model 4) and jointly adjusting for them (model 5).

Conditional multinomial logistic regression models estimating adjusted odds ratios (aORs) were used to examine the associations between diagnosis with any psychiatric disorder and subjection to and perpetration of violence status,^21^ which was defined as an unordered categorical variable with the following categories: (1) neither subjected to violence nor perpetrated violence, (2) subjected to violence only, (3) perpetrated violence only, and (4) both subjected to violence and perpetrated violence. Sensitivity tests for alternative measurement definitions and model specifications were also performed (eMethods in the Supplement). Data were analyzed from January 15 to September 14, 2019.

We accounted for measured confounders, including birth order, parental background factors (eg, low income, low educational level, immigrant background, and history of psychiatric disorders and violent criminality), and the individual’s history of subjection to and perpetration of violence (definitions in eMethods in the Supplement).

## Results

The patient sample comprised 250 419 individuals, of which 138 622 (55.4%) were women and 111 797 (44.6%) were men. The patients were individually matched with 10 people in the general population without psychiatric disorders (n = 2 504 190) and with their siblings without psychiatric disorders (n = 194 788). The largest patient groups included those diagnosed with depression (n = 103 814) or alcohol use disorder (n = 69 116; Table). The median (interquartile range) age at first diagnosis ranged from 20.0 (17.4-24.0) years for alcohol use disorder to 23.7 (19.9-28.8) years for anxiety disorder (Table). The participants had mean (SD) of 7.3 (4.5) years of postdischarge data available.

**Table.** Participant Characteristics by Psychiatric Diagnosis

Characteristic	No. (%)							
	Participants Without Psychiatric Disorder Diagnosis	Participants With Psychiatric Disorder Diagnosis						
		Anxiety	Depression	Bipolar Disorder	Schizophrenia	Personality Disorder	Alcohol Use Disorder	Drug Use Disorder
Total, No.	2 504 190	68 244	103 814	17 309	4153	29 713	69 116	37 039
Age at first diagnosis, median (IQR), y	NA	23.7 (19.9-28.8)	23.1 (19.2-28.2 )	22.9 (19.3-27.9)	22.5 (19.4-26.6)	21.8 (18.9-26.1)	20.0 (17.4-24.0)	21.3 (18.7-24.9)
Sex								
Male	1 117 970 (44.6)	27 787 (40.7)	39 448 (38.0)	5844 (33.8)	2753 (66.3)	10 676 (35.9)	38 987 (56.4)	24 161 (65.2)
Female	1 386 220 (55.4)	40 457 (59.3)	64 366 (62.0)	11 465 (66.2)	1400 (33.7)	19 037 (64.1)	30 129 (43.6)	12 878 (34.8)
Birth order								
1	1 036 966 (41.4)	28 405 (41.6)	42 922 (41.3)	7438 (43.0)	1696 (40.8)	12 571 (42.3)	27 383 (39.6)	15 270 (41.2)
2	923 286 (36.9)	24 094 (35.3)	36 417 (35.1)	5926 (34.2)	1557 (37.5)	9926 (33.4)	24 931 (36.1)	12 924 (34.9)
3	394 206 (15.7)	10 680 (15.6)	16 712 (16.1)	2720 (15.7)	575 (13.8)	4767 (16.0)	11 415 (16.5)	5899 (15.9)
≥4	149 732 (6.0)	5065 (7.4)	7763 (7.5)	1225 (7.1)	325 (7.8)	2449 (8.2)	5387 (7.8)	2946 (8.0)
Immigrant background								
No	2 280 324 (91.1)	61 158 (89.6)	93 885 (90.4)	15683 (90.6)	3551 (85.5)	26 590 (89.5)	62 708 (90.7)	31 955 (86.3)
Yes	223 866 (8.9)	7086 (10.4)	9929 (9.6)	1626 (9.4)	602 (14.5)	3123 (10.5)	6408 (9.3)	5084 (13.7)
Parental income in bottom decile								
No	2 273 931 (90.8)	58 879 (86.3)	89 318 (86.0)	14 708 (85.0)	3247 (78.2)	24 236 (81.6)	58 960 (85.3)	29 659 (80.1)
Yes	230 259 (9.2)	9365 (13.7)	14496 (14.0)	2601 (15.0)	906 (21.8)	5477 (18.4)	10 156 (14.7)	7380 (19.9)
Low parental education level								
No	2 323 875 (92.8)	62 116 (91.0)	95 145 (91.6)	16 020 (92.6)	3727 (89.7)	26 820 (90.3)	62 947 (91.1)	33 018 (89.1)
Yes	180 315 (7.2)	6128 (9.0)	8669 (8.4)	1289 (7.4)	426 (10.3)	2893 (9.7)	6169 (8.9)	4021 (10.9)
Parental lifetime violent crime conviction								
No	2 349 118 (93.8)	60 514 (88.7)	91 719 (88.3)	15 272 (88.2)	3595 (86.6)	25 026 (84.2)	58 678 (84.9)	29 158 (78.7)
Yes	155 072 (6.2)	7730 (11.3)	12 095 (11.7)	2037 (11.8)	558 (13.4)	4687 (15.8)	10 438 (15.1)	7881 (21.3)
Parental lifetime psychiatric morbidity								
No	1 920 673 (76.7)	42 344 (62.0)	62 145 (59.9)	9606 (55.5)	2305 (55.5)	16 226 (54.6)	41 781 (60.5)	19 132 (51.7)
Yes	583 517 (23.3)	25 900 (38.0)	41 669 (40.1)	7703 (44.5)	1848 (44.5)	13 487 (45.4)	27 335 (39.5)	17 907 (48.3)
History of violence								
None	2 442 123 (97.5)	62 987 (92.3)	96 599 (93.1)	16 123 (93.1)	3724 (89.7)	26 444 (89.0)	60 643 (87.7)	28 351 (76.5)
Subjected to violence only	28 305 (1.1)	1966 (2.9)	2856 (2.8)	513 (3.0)	77 (1.9)	959 (3.2)	2960 (4.3)	1934 (5.2)
Perpetrated violence only	30 279 (1.2)	2774 (4.1)	3714 (3.6)	578 (3.3)	317 (7.6)	1982 (6.7)	4518 (6.5)	5677 (15.3)
Both subjected to violence and perpetrated violence	3483 (0.1)	517 (0.8)	645 (0.6)	95 (0.5)	35 (0.8)	328 (1.1)	995 (1.4)	1077 (2.9)

Abbreviation: IQR, interquartile range.

**Figure 1.**
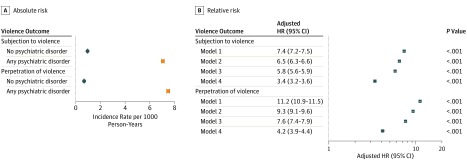
Risk of Subjection to Violence and Perpetration of Violence Among Individuals Diagnosed With Any Psychiatric Disorder Compared With Individuals Without a Psychiatric Disorder Model 1 included matches by sex and birth year. Model 2 was adjusted for birth order and parental characteristics (immigrant background, low income, low educational level, lifetime violent crime conviction, and psychiatric history). Model 3 was further adjusted for the individual’s history of subjection to and perpetration of violence. Model 4 included within-family estimates comparing differentially exposed siblings and adjusted for sex, birth year, birth order, and the individual’s history of subjection to and perpetration of violence. Because the comparisons were made within families, there was no need to adjust for factors that were constant within families. HR indicates hazard ratio.

Less than half of the individuals who were diagnosed with any psychiatric disorder were either subjected to violence to the extent that they required medical treatment or were convicted of a violent crime after the onset of their condition. The unadjusted incidence rates were similar between both outcomes (7.1 [95% CI, 6.9-7.2] vs 7.5 [95% CI, 7.4-7.6] per 1000 person-years among patients diagnosed with psychiatric disorders and 1.0 [95% CI, 0.9-1.0] vs 0.7 [95% CI, 0.7-0.7] per 1000 person-years among individuals without psychiatric disorders) (Figure 1A). The 10-year cumulative incidence rates adjusted for sex and birth year were also similar, ranging from 6.4% to 6.5% among patients with psychiatric disorders and from 0.9% to 0.6% among individuals without psychiatric disorders (eFigures 1 and 2 in the Supplement). In addition, we found that the outcomes co-occurred to a moderate extent among patients with psychiatric disorders (*r* = 0.43 [95% CI, 0.41-0.45]).

We initially found that persons who were diagnosed with any psychiatric disorder were more than 7 times as likely as those without psychiatric disorders to be subjected to violence (aHR, 7.4 [95% CI, 7.2-7.5]; model 1 in Figure 1B). Further adjustments for birth order and parental confounders (model 2) and the individual’s history of subjection to violence and perpetration of violence (model 3) attenuated those estimates to a nearly 6-fold risk increase (aHR, 5.8 [95% CI, 5.6-5.9]). Unmeasured familial confounders were important because the sibling comparison estimate (model 4) further attenuated the association to an approximately 3-fold risk increase (aHR, 3.4 [95% CI, 3.2-3.6]). We observed a similar pattern of associations for violent perpetration as the outcome, ranging from an 11-fold risk increase in the crude model (aHR, 11.2 [95% CI, 10.9-11.5]) to a 4-fold risk increase in the fully adjusted model (aHR, 4.2 [95% CI, 3.9-4.4]). We found the model 4 estimates to be robust to most model specifications, with effect sizes typically ranging from a 3- to 4-fold risk increase across both of the outcomes (eFigure 3 in the Supplement).

**Figure 2.**
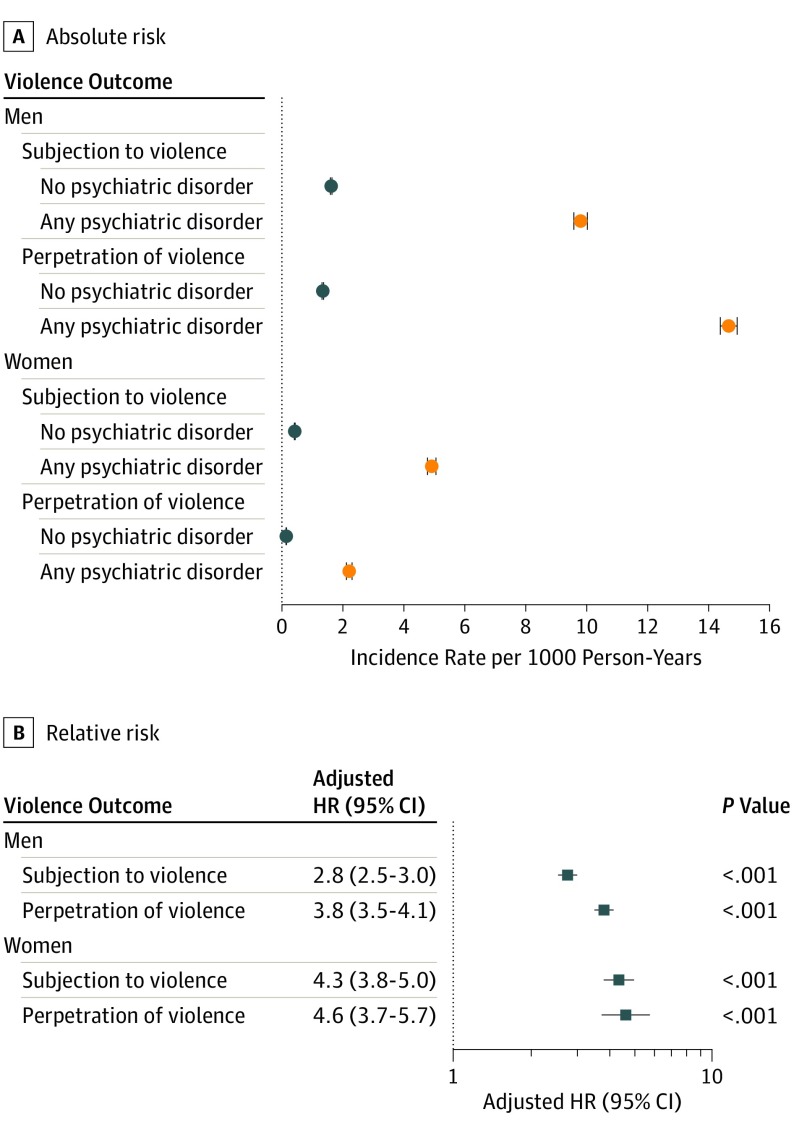
Sex-Stratified Risk of Subjection to Violence and Perpetration of Violence Among Individuals Diagnosed With Any Psychiatric Disorder Compared With Siblings Without Psychiatric Disorders The adjusted hazard ratios refer to within-family estimates comparing differentially exposed siblings and adjusted for sex, birth year, birth order, and the individual’s history of subjection to and perpetration of violence. Because the comparisons were made within families, there was no need to adjust for factors that were constant within families. HR indicates hazard ratio.

**Figure 3.**
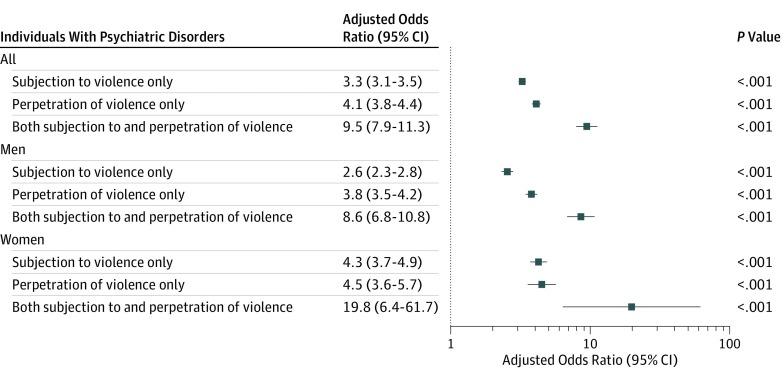
Adjusted Odds Ratios for Subjection to Violence Only, Perpetration of Violence Only, and Both Subjection to and Perpetration of Violence Among Men and Women With Psychiatric Disorders The adjusted odds ratios refer to within-family estimates comparing differentially exposed siblings and adjusted for sex, birth year, birth order, and the individual’s history of subjection to and perpetration of violence. Because the comparisons were made within families, there was no need to adjust for factors that were constant within families.

We observed sex differences in the distribution of the violence outcomes (Figure 2A). Men with any psychiatric disorder were approximately 3 times more likely to be subjected to violence (aHR, 2.8 [95% CI, 2.5-3.0]) and approximately 4 times more likely to perpetrate violence (aHR, 3.8 [95% CI, 3.5-4.1]; Figure 2B) than their siblings without psychiatric disorders. We observed minor differences for the equivalent estimates in women among those who were subjected to violence (aHR, 4.3 [95% CI, 3.8-5.0]) and among those who perpetrated violence (aHR, 4.6 [95% CI, 3.7-5.7]) (Figure 2B). Assuming that the violence outcomes were directly comparable, we observed that both men and women with any psychiatric disorder were more likely than their siblings without psychiatric disorders to be both subjected to violence and to perpetrate violence (aOR, men, 8.6 [95% CI, 6.8-10.8]; aOR, women, 19.8 [95% CI, 6.4-61.7]) than to have solely experienced subjection to violence (aOR, men, 2.5 [95% CI, 2.3-2.8]; aOR, women, 4.3 [95% CI, 3.7-4.9]) or to have solely perpetrated violence (aOR, men, 3.8 [95% CI, 3.4-4.2]; aOR, women, 4.5 [95% CI, 3.7-4.9]; Figure 3).

**Figure 4.**
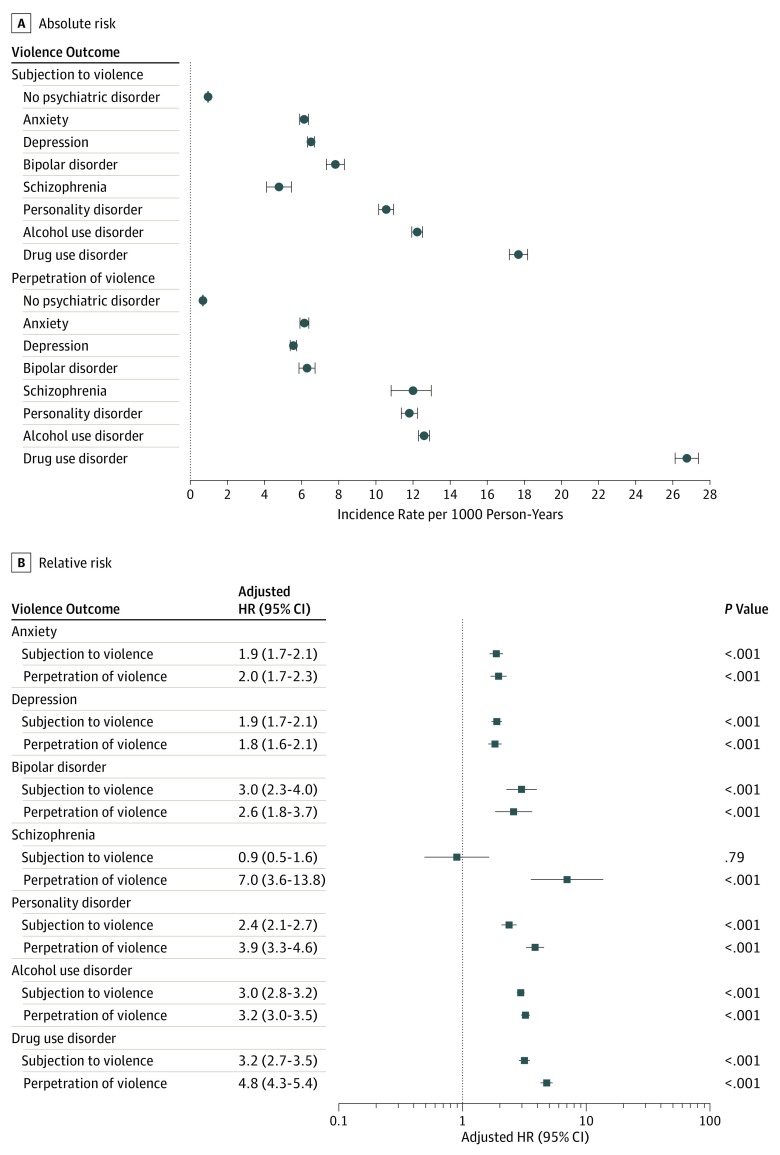
Risk of Subjection to Violence and Perpetration of Violence Among Individuals Diagnosed With Specific Psychiatric Disorders Compared With Siblings Without Psychiatric Disorders The adjusted hazard ratios refer to within-family estimates comparing differentially exposed siblings and adjusted for sex, birth year, birth order, and the individual’s history of subjection to and perpetration of violence. The estimates were further jointly adjusted for all of the psychiatric disorders and substance use disorders. Because the comparisons were made within families, there was no need to adjust for factors that were constant within families. HR indicates hazard ratio.

Further stratification by psychiatric diagnoses indicated that the outcomes were more common among persons diagnosed with drug and alcohol use disorders (12-17 subjection to violence events and 13-27 perpetration of violence events per 1000 person-years) than in other diagnostic groups (Figure 4A). We initially found that persons with any of the specific psychiatric disorders were more likely than their siblings without psychiatric disorders to be subjected to violence (range between aHR, 2.3 [95% CI, 1.5-3.5] to aHR, 5.3 [95% CI, 4.3-6.7], respectively) and to perpetrate violence against others (range between aHR, 3.1 [95% CI, 2.7-3.6] to aHR, 9.6 [95% CI, 5.6-16.6], respectively; model 4 in eFigure 4 in the Supplement). However, when we jointly adjusted for all of the conditions, we found that these estimates were attenuated but remained statistically significant for both outcomes, with the sole exception of persons diagnosed with schizophrenia, who did not have a higher risk of being subjected to violence when compared with their siblings without psychiatric disorders (aHR, 0.9 [95% CI, 0.5-1.6]; Figure 4).

## Discussion

In this nationwide study of 250 419 individuals born between 1973 and 1993 in Sweden, we examined the associations between psychiatric disorders and the later risk of subjection to violence and perpetration of violence. The patients were matched by age and sex to a general population control group and to their full biological siblings without psychiatric disorders. To our knowledge, this is the first study to have examined these associations using a sibling comparison approach, which enabled us to account for important shared unmeasured familial (eg, genetic and environmental) confounders. Our study had 4 principal findings.

First, we estimated that the 10-year cumulative incidence rate of being subjected to violence was less than 7% in persons diagnosed with any psychiatric disorder. This estimate is therefore considerably smaller in magnitude, even when compared with the previous annual rates for subjection to violence reported in studies from Sweden,^24^ the Netherlands,^25^ the United Kingdom,^13^ and the United States,^26,27^ which typically range between 20% and 60%. This discrepancy is expected, as the earlier research relied on broad and self-reported measures of subjection to violence and were based on selected samples.

Second, associations between psychiatric morbidity and a later risk of subjection to violence were considerably attenuated once we accounted for the individual’s history of subjection to and perpetration of violence as well as for unmeasured familial confounding by comparing patients with psychiatric conditions with their siblings without psychiatric disorders. The estimated risk increase of subjection to violence among people with psychiatric diagnoses was reduced from a factor of 6.5 to 3.4. These findings suggest that the estimates reported in 2 previous population studies, which examined alternative subjection to violence outcomes (eg, police-reported events^16^ and homicidal deaths^15^), may have been substantially overestimated because of the lack of adjustment for these factors.

Third, we found that the risks of subjection to and perpetration of violence varied across specific psychiatric disorders and were highest in persons with substance use disorders. In contrast, after adjusting for comorbid substance use disorders and personality disorders, we observed that persons diagnosed with schizophrenia were no more likely than their siblings without psychiatric disorders to be subjected to violence. One explanation for this finding is that patients with schizophrenia who do not have the comorbid conditions are more socially isolated and therefore less likely to be in environments where the risk of subjection to violence is increased.

Fourth, consistent with the literature,^28,29,30,31^ we found overlap between the risk of subjection to violence and perpetration of violence in individuals with psychiatric disorders. Although direct comparisons of the outcome measures require cautious interpretation, we note that this overlap may be important because it may offer etiologic and treatment targets. By separately considering each outcome, the dynamic interplay between them is overlooked. To take one example, subjection to violence is a trigger for subsequent perpetration of violence in patients diagnosed with psychotic disorders and individuals without psychiatric disorders.^32^

Our findings are largely consistent with those of the MacArthur risk assessment study, which found elevated postdischarge rates of violence among patients with psychiatric illnesses and comorbid substance use disorders as well as among individuals with early experiences of physical abuse and violence perpetration.^33^ Our findings diverge with regard to the association between certain psychiatric disorders and violence perpetration; we found higher rates of perpetration among persons with schizophrenia than depression.^33^ These observed differences could potentially be associated with contextual differences between Sweden and the United States, but we note that our study had sufficient statistical power to estimate differences between the conditions with a high degree of precision (250 419 vs 951 patients).^33^ Our study also benefited from almost no selection bias, unlike clinical studies in which the nonconsenting patients may have had different background risks. In the MacArthur study, for example, 44% of nonconsenting patients had schizophrenia diagnoses and were more likely than consenting patients to have histories of violence.^33^

### Strengths and Limitations

Our study has several strengths. The use of Swedish national registers allowed us to study more than 250 000 patients diagnosed with psychiatric disorders, each individually matched with 10 people in the general population, while keeping selection bias to a minimum, as more than 95% of the overall sample was retained. We defined subjection to violence, using objective and validated measures of assault, as being events that either required hospital care (including specialist outpatient visits but excluding primary care visits) or resulted in death, in a country with universal health care. By adopting the sibling comparison approach, we were able to control for unmeasured familial confounding for the first time, to our knowledge.

However, the study had important limitations. First, we did not include incidents of less severe subjection to violence that did not result in hospitalization or death, which suggests that the reported absolute risk estimates should be interpreted as capturing the most severe violence subjection events. At the same time, our measures had the advantage of focusing on patients for whom implications for clinical services existed and interventions were potentially available (as these patients would have had service contacts). The extent to which the magnitude of the examined associations differs between severity levels remains relatively unknown and needs to be addressed in future studies. We would, however, expect that the inclusion of violence subjection events with a lower severity level would attenuate the reported relative risk estimates. This expectation is consistent with our sensitivity analysis, which observed a dose-response association between psychiatric morbidity and the severity of violence subjection events, a finding also reported in previous studies.^15,16^ Furthermore, it has been reported that approaches to measuring less severe violence subjection events (eg, self-reports and police reports) are associated with substantial measurement error, particularly when studying adolescents and individuals with elevated levels of psychiatric symptoms.^34,35^ Notably, the combination of using self-reports and sibling comparisons would further inflate the measurement error, causing an artificial bias of the estimates toward the null.^36^

Second, although sibling comparisons offer a powerful approach that accounts for genetic confounding, they account for approximately half of the genetic influences. Given the large reductions of the estimates in the sibling comparison models, we have likely overestimated the true associations.

Third, nationwide registries lack sufficient detail to fully ascertain the timing of our measures. Future studies may benefit from combining developmental life-course approaches with quasi-experimental designs to assess the relative importance of timing effects (eg, changes to diagnoses over time) and etiologic mechanisms (eg, mediation and moderation effects). Fourth, we had an mean of 7.3 years of postdischarge data available per participant, which captured a limited portion of their lives. Although our data were similar in magnitude to those of related Scandinavian population-based studies that had a maximum of 8 to 13 years of follow-up data,^15,16^ a need exists for studies with longer follow-up data to improve understanding of the long-term developmental trajectories of violent outcomes in people with psychiatric disorders. Fifth, although we used similar definitions for our outcome measures, they were derived from different data sources, which implies that comparisons between them should be interpreted with caution. Subjection to violence measures potentially represent a higher threshold because they require individuals to access health care services, although this assertion requires more empirical evidence to test. We note that the correlation between the outcomes in our patient sample (*r* = 0.43 [95% CI, 0.41-0.45]) replicated that of the MacArthur study (*r* = 0.44 [95% CI, 0.32-0.56]).^37^ Their data have been widely used to examine the co-occurrence between the outcomes despite heterogeneous definitions and data collection strategies.^21,27,38^

Sixth, the generalizability of our findings is unclear. Internationally comparable surveys of individuals subjected to violent crime reported that the annual rate of subjection to violence in Sweden (3.5%) was comparable with the global average (3.1%).^39^ Furthermore, a 2010 systematic review did not observe any clear differences in the rates of psychiatric disorders across Western European countries.^40^ However, associations between psychiatric disorders and violence outcomes may vary in other contexts, particularly in countries with different base rates of violence. Future studies should therefore test for this variance by using large-scale population-based data with adjustments for unmeasured familial confounders and early experiences of violence.

## Conclusions

In this large longitudinal cohort study, we found that individuals diagnosed with psychiatric disorders in Sweden were more likely than 2 comparison groups without psychiatric disorders—siblings and individuals of similar age and gender in the general population—to be subjected to violence and to perpetrate violence against others. We generally found the magnitude of the associations to be similar across both outcomes, indicating a 3- to 4-fold elevated risk when the patients were compared with siblings who did not have psychiatric disorders. In addition, we found that having a diagnosis of schizophrenia was not associated with subsequent subjection to violence after we accounted for comorbid substance use and personality disorders. In contrast, we found that the same condition was the strongest risk factor for the perpetration of violence. Our findings underscore the need to address comorbid substance use and personality disorders to develop scalable approaches that assess and manage the risk of subjection to and perpetration of violence in people with psychiatric disorders.

## Notes

Supplement. **eMethods.** Population Sample, Definitions of Measured Confounders, Validation of Diagnoses, Complementary Sensitivity Analyses, and Moderation Effects **eTable 1**. STROBE Statement **eTable 2.** *ICD* Codes **eFigure 1.** Sex and Age-Adjusted Cumulative Incidence Rates of Subjection to Violence Across the First 10 Years After the Onset of Any Psychiatric Disorder in Patients Compared With Unaffected Siblings and General Population Control Groups **eFigure 2.** Sex and Age-Adjusted Cumulative Incidence Rates of Perpetration of Violence Across the First 10 Years After the Onset of Any Psychiatric Disorder in Patients Compared With Unaffected Siblings and General Population Control Groups **eFigure 3.** Sensitivity Tests: Alternative Exposure and Outcome Definitions and Matching Criteria **eFigure 4.** Sensitivity Test: Associations Between Specific Psychiatric Disorders and Subsequent Risks of Subjection to Violence and Perpetration of Violence With and Without Adjustments for All Other Psychiatric Disorders **eReferences.**

## References

[1] Chesney E, Goodwin GM, Fazel S Risks of all-cause and suicide mortality in mental disorders: a meta-review. World Psychiatry. 2014;13(2):153-160. doi:10.1002/wps.20128 24890068 PMC4102288

[2] Marwaha S, Johnson S, Bebbington P, Rates and correlates of employment in people with schizophrenia in the UK, France and Germany. Br J Psychiatry. 2007;191:30-37. doi:10.1192/bjp.bp.105.020982 17602122

[3] Nielsen SF, Hjorthoj CR, Erlangsen A, Nordentoft M Psychiatric disorders and mortality among people in homeless shelters in Denmark: a nationwide register-based cohort study. Lancet. 2011;377(9784):2205-2214. doi:10.1016/S0140-6736(11)60747-2 21676456

[4] Stevens H, Laursen TM, Mortensen PB, Agerbo E, Dean K Post-illness-onset risk of offending across the full spectrum of psychiatric disorders. Psychol Med. 2015;45(11):2447-2457. doi:10.1017/S0033291715000458 25851504

[5] Sariaslan A, Larsson H, Fazel S Genetic and environmental determinants of violence risk in psychotic disorders: a multivariate quantitative genetic study of 1.8 million Swedish twins and siblings. Mol Psychiatry. 2016;21(9):1251-1256. doi:10.1038/mp.2015.184 26666206 PMC4842006

[6] Fazel S, Smith EN, Chang Z, Geddes JR Risk factors for interpersonal violence: an umbrella review of meta-analyses. Br J Psychiatry. 2018;213(4):609-614. doi:10.1192/bjp.2018.145 30058516 PMC6157722

[7] Treatment Advocacy Center Victimization and serious mental illness. Treatment Advocacy Center website. https://www.treatmentadvocacycenter.org/evidence-and-research/learn-more-about/3630-victimization-and-serious-mental-illness. Published June 2016. Accessed February 2019.

[8] Pettitt B, Greenhead S, Khalifeh H, At risk, yet dismissed: the criminal victimisation of people with mental health problems. London, UK: Victim Support and Mind; 2013.

[9] Maniglio R Severe mental illness and criminal victimization: a systematic review. Acta Psychiatr Scand. 2009;119(3):180-191. doi:10.1111/j.1600-0447.2008.01300.x 19016668

[10] Khalifeh H Violence against people with severe mental illness in Europe. Acta Psychiatr Scand. 2009;119(5):414. doi:10.1111/j.1600-0447.2009.01374.x 19291079

[11] de Vries B, van Busschbach JT, van der Stouwe ECD, Prevalence rate and risk factors of victimization in adult patients with a psychotic disorder: a systematic review and meta-analysis. Schizophr Bull. 2019;45(1):114-126. doi:10.1093/schbul/sby020 29547958 PMC6293237

[12] Hughes K, Bellis MA, Jones L, Prevalence and risk of violence against adults with disabilities: a systematic review and meta-analysis of observational studies. Lancet. 2012;379(9826):1621-1629. doi:10.1016/S0140-6736(11)61851-5 22377290

[13] Khalifeh H, Johnson S, Howard LM, Violent and non-violent crime against adults with severe mental illness. Br J Psychiatry. 2015;206(4):275-282. doi:10.1192/bjp.bp.114.147843 25698767

[14] Bhavsar V, Bhugra D Violence towards people with mental illness: assessment, risk factors, and management. Psychiatry Clin Neurosci. 2018;72(11):811-820. doi:10.1111/pcn.12775 30084514

[15] Crump C, Sundquist K, Winkleby MA, Sundquist J Mental disorders and vulnerability to homicidal death: Swedish nationwide cohort study. BMJ. 2013;346:f557. doi:10.1136/bmj.f557 23462204 PMC6364268

[16] Dean K, Laursen TM, Pedersen CB, Webb RT, Mortensen PB, Agerbo E Risk of being subjected to crime, including violent crime, after onset of mental illness: a Danish national registry study using police data. JAMA Psychiatry. 2018;75(7):689-696. doi:10.1001/jamapsychiatry.2018.0534 29799904 PMC6071849

[17] Carr CP, Martins CMS, Stingel AM, Lemgruber VB, Juruena MF The role of early life stress in adult psychiatric disorders: a systematic review according to childhood trauma subtypes. J Nerv Ment Dis. 2013;201(12):1007-1020. doi:10.1097/NMD.0000000000000049 24284634

[18] Read J, van Os J, Morrison AP, Ross CA Childhood trauma, psychosis and schizophrenia: a literature review with theoretical and clinical implications. Acta Psychiatr Scand. 2005;112(5):330-350. doi:10.1111/j.1600-0447.2005.00634.x 16223421

[19] Barnes JC, Beaver KM Extending research on the victim-offender overlap: evidence from a genetically informative analysis. J Interpers Violence. 2012;27(16):3299-3321. doi:10.1177/0886260512441259 22585116

[20] Beckley AL, Caspi A, Arseneault L, The developmental nature of the victim-offender overlap. J Dev Life Course Criminol. 2018;4(1):24-49. doi:10.1007/s40865-017-0068-3 29581934 PMC5865449

[21] Johnson KL, Desmarais SL, Van Dorn RA, Grimm KJ A typology of community violence perpetration and victimization among adults with mental illnesses. J Interpers Violence. 2015;30(3):522-540. doi:10.1177/0886260514535102 24919996 PMC4263811

[22] Ludvigsson JF, Almqvist C, Bonamy AK, Registers of the Swedish total population and their use in medical research. Eur J Epidemiol. 2016;31(2):125-136. doi:10.1007/s10654-016-0117-y 26769609

[23] Ludvigsson JF, Haberg SE, Knudsen GP, Ethical aspects of registry-based research in the Nordic countries. Clin Epidemiol. 2015;7:491-508. doi:10.2147/CLEP.S90589 26648756 PMC4664438

[24] Sturup J, Sorman K, Lindqvist P, Kristiansson M Violent victimization of psychiatric patients: a Swedish case-control study. Soc Psychiatry Psychiatr Epidemiol. 2011;46(1):29-34. doi:10.1007/s00127-009-0167-5 19916061 PMC3024491

[25] Kamperman AM, Henrichs J, Bogaerts S, Criminal victimisation in people with severe mental illness: a multi-site prevalence and incidence survey in the Netherlands. PLoS One. 2014;9(3):e91029. doi:10.1371/journal.pone.0091029 24609108 PMC3946683

[26] Teplin LA, McClelland GM, Abram KM, Weiner DA Crime victimization in adults with severe mental illness: comparison with the National Crime Victimization Survey. Arch Gen Psychiatry. 2005;62(8):911-921. doi:10.1001/archpsyc.62.8.911 16061769 PMC1389236

[27] Desmarais SL, Van Dorn RA, Johnson KL, Grimm KJ, Douglas KS, Swartz MS Community violence perpetration and victimization among adults with mental illnesses. Am J Public Health. 2014;104(12):2342-2349. doi:10.2105/AJPH.2013.301680 24524530 PMC4133297

[28] Hiday VA, Swartz MS, Swanson JW, Borum R, Wagner HR Criminal victimization of persons with severe mental illness. Psychiatr Serv. 1999;50(1):62-68. doi:10.1176/ps.50.1.62 9890581

[29] Hiday VA, Swanson JW, Swartz MS, Borum R, Wagner HR Victimization: a link between mental illness and violence? Int J Law Psychiatry. 2001;24(6):559-572. doi:10.1016/S0160-2527(01)00091-7 11795220

[30] Sadeh N, Binder RL, McNiel DE Recent victimization increases risk for violence in justice-involved persons with mental illness. Law Hum Behav. 2014;38(2):119-125. doi:10.1037/lhb0000043 23855324

[31] Johnson KL, Desmarais SL, Tueller SJ, Grimm KJ, Swartz MS, Van Dorn RA A longitudinal analysis of the overlap between violence and victimization among adults with mental illnesses. Psychiatry Res. 2016;246:203-210. doi:10.1016/j.psychres.2016.09.039 27721058 PMC5161544

[32] Sariaslan A, Lichtenstein P, Larsson H, Fazel S Triggers for violent criminality in patients with psychotic disorders. JAMA Psychiatry. 2016;73(8):796-803. doi:10.1001/jamapsychiatry.2016.1349 27410165 PMC5047356

[33] Monahan J, Steadman HJ, Silver E, Rethinking Risk Assessment: the MacArthur Study of Mental Disorder and Violence. New York: Oxford University Press; 2001.

[34] van Giezen AE, Arensman E, Spinhoven P, Wolters G Consistency of memory for emotionally arousing events: a review of prospective and experimental studies. Clin Psychol Rev. 2005;25(7):935-953. doi:10.1016/j.cpr.2005.04.011 15979220

[35] Mesquita CS, Maia AC What is told when the story is retold? consistency of victimization reports in psychiatric patients. Scand J Psychol. 2018;59(3):311-318. doi:10.1111/sjop.12437 29533460

[36] Frisell T, Oberg S, Kuja-Halkola R, Sjolander A Sibling comparison designs: bias from non-shared confounders and measurement error. Epidemiology. 2012;23(5):713-720. doi:10.1097/EDE.0b013e31825fa230 22781362

[37] Silver E, Piquero AR, Jennings WG, Piquero NL, Leiber M Assessing the violent offending and violent victimization overlap among discharged psychiatric patients. Law Hum Behav. 2011;35(1):49-59. doi:10.1007/s10979-009-9206-8 20145985

[38] Van Dorn RA, Grimm KJ, Desmarais SL, Tueller SJ, Johnson KL, Swartz MS Leading indicators of community-based violent events among adults with mental illness. Psychol Med. 2017;47(7):1179-1191. doi:10.1017/S0033291716003160 27998319

[39] van Dijk J, van Kestere J, Smit P Criminal Victimisation in International Perspective: Key Findings from the 2004-2005 ICVS and EU ICS. The Hague: United Nations Office on Drugs and Crime and United Nations Interregional Crime; 2008.

[40] Wittchen HU, Jacobi F, Rehm J, The size and burden of mental disorders and other disorders of the brain in Europe 2010. Eur Neuropsychopharmacol. 2011;21(9):655-679. doi:10.1016/j.euroneuro.2011.07.018 21896369

