# Supplementary material for: Risk of Subjection to Violence and Perpetration of Violence in Persons With Psychiatric Disorders in Sweden
Source: JAMA Psychiatry. 2020 Jan 15;77(4):359–67. doi: 10.1001/jamapsychiatry.2019.4275 (PMC6990843; doi:10.1001/jamapsychiatry.2019.4275)
Supplement: Supplement. — eMethods. Population Sample, Definitions of Measured Confounders, Validation of Diagnoses, Complementary Sensitivity Analyses, and Moderation Effects eTable 1. STROBE Statement eTable 2. ICD Codes eFigure 1. Sex and Age-Adjusted Cumulative Incidence Rates of Subjection to Violence Across the First 10 Years After the Onset of Any Psychiatric Disorder in Patients Compared With Unaffected Siblings and General Population Control Groups eFigure 2. Sex and Age-Adjusted Cumulative Incidence Rates of Perpetration of Violence Across the First 10 Years After the Onset of Any Psychiatric Disorder in Patients Compared With Unaffected Siblings and General Population Control Groups eFigure 3. Sensitivity Tests: Alternative Exposure and Outcome Definitions and Matching Criteria eFigure 4. Sensitivity Test: Associations Between Specific Psychiatric Disorders and Subsequent Risks of Subjection to Violence and Perpetration of Violence With and Without Adjustments for All Other Psychiatric Disorders eReferences. [file jamapsychiatry-77-359-s001.pdf]

Supplementary Online Content

Sariaslan A, Arseneault L, Larsson H, Lichtenstein P, Fazel S. Risk of subjection to violence and perpetration of violence in persons with psychiatric disorders in Sweden. *JAMA Psychiatry*. Published January 15, 2020. doi:10.1001/jamapsychiatry.2019.4275

eMethods. Population Sample, Definitions of Measured Confounders, Validation of Diagnoses, Complementary Sensitivity Analyses, and Moderation Effects

eTable 1. STROBE Statement

eTable 2. *ICD* Codes

eFigure 1. Sex and Age-Adjusted Cumulative Incidence Rates of Subjection to Violence Across the First 10 Years After the Onset of Any Psychiatric Disorder in Patients Compared With Unaffected Siblings and General Population Control Groups

eFigure 2. Sex and Age-Adjusted Cumulative Incidence Rates of Perpetration of Violence Across the First 10 Years After the Onset of Any Psychiatric Disorder in Patients Compared With Unaffected Siblings and General Population Control Groups

eFigure 3. Sensitivity Tests: Alternative Exposure and Outcome Definitions and Matching Criteria

eFigure 4. Sensitivity Test: Associations Between Specific Psychiatric Disorders and Subsequent Risks of Subjection to Violence and Perpetration of Violence With and Without Adjustments for All Other Psychiatric Disorders

eReferences.

This supplementary material has been provided by the authors to give readers additional information about their work.

© 2020 Sariaslan A et al. *JAMA Psychiatry*

eMethods. Population Sample, Definitions of Measured Confounders, Validation of Diagnoses, Complementary Sensitivity Analyses, and Moderation Effects

Population Sample

Our original sample consisted of all children born in Sweden 1973-1993 who could be linked to both of their biological parents (n=2,176,150). We chose these years as they captured information on all psychiatric disorders and covariates (as the National Patient and Crime registers were available from 1973) and also on outcomes (as the legal age of responsibility in Sweden is 15 years and we wanted the youngest cohorts to have sufficient time to have outcomes). We excluded those who had emigrated (n=73,717) or died (n=17,420) before age 15 as well as those who lacked data on parental socioeconomic measures (n=5149), thus resulting in a final population size of 2,079,864 individuals (95.6% of the targeted population). All of the patients and controls were selected from this sample.

Definitions of Measured Confounders

Immigrant status was defined as having at least one biological parent who was born in a non-Nordic country. Parental history of psychiatric morbidity and violent criminality indicated whether either biological parent had been diagnosed for any psychiatric disorder or had a violent criminal conviction. Low family income indicated whether the individual's disposable family income (e.g., standardized net sum of earnings and benefits averaged across both biological parents) measured at the end of the year that they turned 15 years of age ranked in the bottom decile of the population. If this information was missing, we used data from the previous year or until it became available. We adopted this non-linear definition because earlier studies have shown that rates of violent convictions are heavily concentrated in the most deprived groups in Sweden.[1,2] Similar findings have also been observed, albeit less pronounced, for violent victimization in Finland.[3,4] Low parental educational attainment indicated that neither biological parent had achieved secondary school qualifications.

The individual's history of violent victimization and perpetration was defined, consistent with the literature,[5] as an unordered categorical variable with the following four categories: (a) neither victim nor perpetrator, (b) victim only, (c) perpetrator only and (d) both victim and perpetrator. The patient data was not used to determine perpetration status and the conviction data was not used to define victim status.

© 2020 Sariaslan A et al. *JAMA Psychiatry*

Validation of Diagnoses

The National Patient Register (NPR) offers a near full coverage (>99%) of all somatic (including surgery) and psychiatric inpatient care discharges since 1973 and specialist outpatient visits since 2001.[6] A 2011 review of the validity of all inpatient care diagnoses reported in the NPR found that the positive predictive values typically ranged between 85-95%.[6] We are unaware of any validation studies that have examined victimization diagnoses specifically but there is some evidence suggesting that the severity levels of the victimization injuries are valid.[7,8] We also note that the violent victimization diagnoses have been used in large-scale epidemiological studies conducted not only in Sweden[9] but also in comparable registers in Finland[4] and Denmark.[10] Validation studies examining NPR hospital admissions for psychiatric and substance use disorders have demonstrated fair to excellent validity (Cohen's kappa>0.70 for schizophrenia and anxiety related disorders; >0.30 for mood and substance use disorders).[7,11–14]

Complementary Sensitivity Analyses

We carried out sensitivity analyses using alternative measurement definitions and model specifications to test for the stability of the fully adjusted co-sibling estimates (Model IV). First, we used a stricter definition of psychiatric disorders by requiring at least two separately occurring episodes and also for violent victimization severity by only considering inpatient care cases of victimization and homicidal deaths. We further tested for the associations by only considering the "core" psychiatric disorders (e.g., anxiety, depression, bipolar disorder and schizophrenia). Second, we tested for alternative matching criteria, either by only including unaffected same-sexed siblings or unaffected siblings of both sexes born within four years of the patients. Third, to test for potential misclassification bias, we excluded individuals who had been diagnosed with having been exposed to an unarmed brawl or fight (ICD-10 code: Y04). Lastly, we tested for violent crime arrests (derived from the National Criminal Suspects Register) instead of violent crime convictions as outcome.

Moderation Effects

We tested for moderation effects by specifying a number of additional statistical models that included interaction terms for the following research questions. First, we asked whether the associations

between being diagnosed with any psychiatric disorder and the violence outcomes were moderated by the individual's history of violent victimization and perpetration. Second, we asked whether the association between being diagnosed with any of the "core" psychiatric disorders (e.g., anxiety, depression, bipolar disorder and schizophrenia) and the violence outcomes were moderated by comorbid personality disorder, alcohol use disorder and/or drug use disorder. There was little evidence of any of the examined moderation effects as none of the interaction terms were statistically significant (all $p>0.05$ following Bonferroni corrections for multiple testing).

© 2020 Sariaslan A et al. *JAMA Psychiatry*

eTable 1. STROBE Statement

	Item No	Recommendation	Page No
**Title and abstract**	1	(*a*) Indicate the study's design with a commonly used term in the title or the abstract	
		(*b*) Provide in the abstract an informative and balanced summary of what was done and what was found	1, 3-4
**Introduction**			
Background/rationale	2	Explain the scientific background and rationale for the investigation being reported	5,6
Objectives	3	State specific objectives, including any prespecified hypotheses	6
**Methods**			
Study design	4	Present key elements of study design early in the paper	6
Setting	5	Describe the setting, locations, and relevant dates, including periods of recruitment, exposure, follow-up, and data collection	6, eMethods
Participants	6	(*a*) Give the eligibility criteria, and the sources and methods of selection of participants. Describe methods of follow-up	
		(*b*) For matched studies, give matching criteria and number of exposed and unexposed	7-8, eMethods
Variables	7	Clearly define all outcomes, exposures, predictors, potential confounders, and effect modifiers. Give diagnostic criteria, if applicable	7-8, eMethods
Data sources/ measurement	8*	For each variable of interest, give sources of data and details of methods of assessment (measurement). Describe comparability of assessment methods if there is more than one group	6-8, eMethods, eTable 2
Bias	9	Describe any efforts to address potential sources of bias	9, eMethods
Study size	10	Explain how the study size was arrived at	7-8, eMethods
Quantitative variables	11	Explain how quantitative variables were handled in the analyses. If applicable, describe which groupings were chosen and why	eMethods
Statistical methods	12	(*a*) Describe all statistical methods, including those used to control for confounding	8-10, eMethods
		(*b*) Describe any methods used to examine subgroups and interactions	
		(*c*) Explain how missing data were addressed	
		(*d*) If applicable, explain how loss to follow-up was addressed	
		(*e*) Describe any sensitivity analyses	

© 2020 Sariaslan A et al. *JAMA Psychiatry*

**Results**			
Participants	13*	(a) Report numbers of individuals at each stage of study—eg numbers potentially eligible, examined for eligibility, confirmed eligible, included in the study, completing follow-up, and analysed	eMethods
		(b) Give reasons for non-participation at each stage	
		(c) Consider use of a flow diagram	
Descriptive data	14*	(a) Give characteristics of study participants (eg demographic, clinical, social) and information on exposures and potential confounders	Table 1, eMethods
		(b) Indicate number of participants with missing data for each variable of interest	
		(c) Summarise follow-up time (eg, average and total amount)	
Outcome data	15*	Report numbers of outcome events or summary measures over time	9-10, eFigures1-2

Main results	16	(*a*) Give unadjusted estimates and, if applicable, confounder-adjusted estimates and their precision (eg, 95% confidence interval). Make clear which confounders were adjusted for and why they were included	9-11
		(*b*) Report category boundaries when continuous variables were categorized	
		(*c*) If relevant, consider translating estimates of relative risk into absolute risk for a meaningful time period	
Other analyses	17	Report other analyses done—eg analyses of subgroups and interactions, and sensitivity analyses	10-11, eFigure 3
**Discussion**			
Key results	18	Summarise key results with reference to study objectives	11-13
Limitations	19	Discuss limitations of the study, taking into account sources of potential bias or imprecision. Discuss both direction and magnitude of any potential bias	13-15
Interpretation	20	Give a cautious overall interpretation of results considering objectives, limitations, multiplicity of analyses, results from similar studies, and other relevant evidence	15-16
Generalisability	21	Discuss the generalisability (external validity) of the study results	15
**Other information**			
Funding	22	Give the source of funding and the role of the funders for the present study and, if applicable, for the original study on which the present article is based	17

*Give information separately for exposed and unexposed groups.

© 2020 Sariaslan A et al. *JAMA Psychiatry*

eTable 2. *ICD* Codes

	**ICD-8 (1969-1986)**	**ICD-9 (1987-1996)**	**ICD-10 (1997-)**
Violent victimization	E960-E969	E960-E969	X85-X99, Y00-Y09
Schizophrenia	295	295	F20
Bipolar disorder	296 except 296.2	296 except 296B	F30-F31
Depression	296.2, 300.4	296B, 300E, 311	F32-F39 except F32.3
Anxiety	300 except 300.4	300 except 300E	F40-F42, F44-F45, F48
Personality disorder	301	301	F60-F69
Alcohol use disorders	291, 303	291, 303, 305A	F10
Drug use disorders	292, 304	292, 304, 305X	F11-12, F14-F16, F19

© 2020 Sariaslan A et al. *JAMA Psychiatry*

eFigure 1. Sex and Age-Adjusted Cumulative Incidence Rates of Subjection to Violence Across the First 10 Years After the Onset of Any Psychiatric Disorder in Patients Compared With Unaffected Siblings and General Population Control Groups

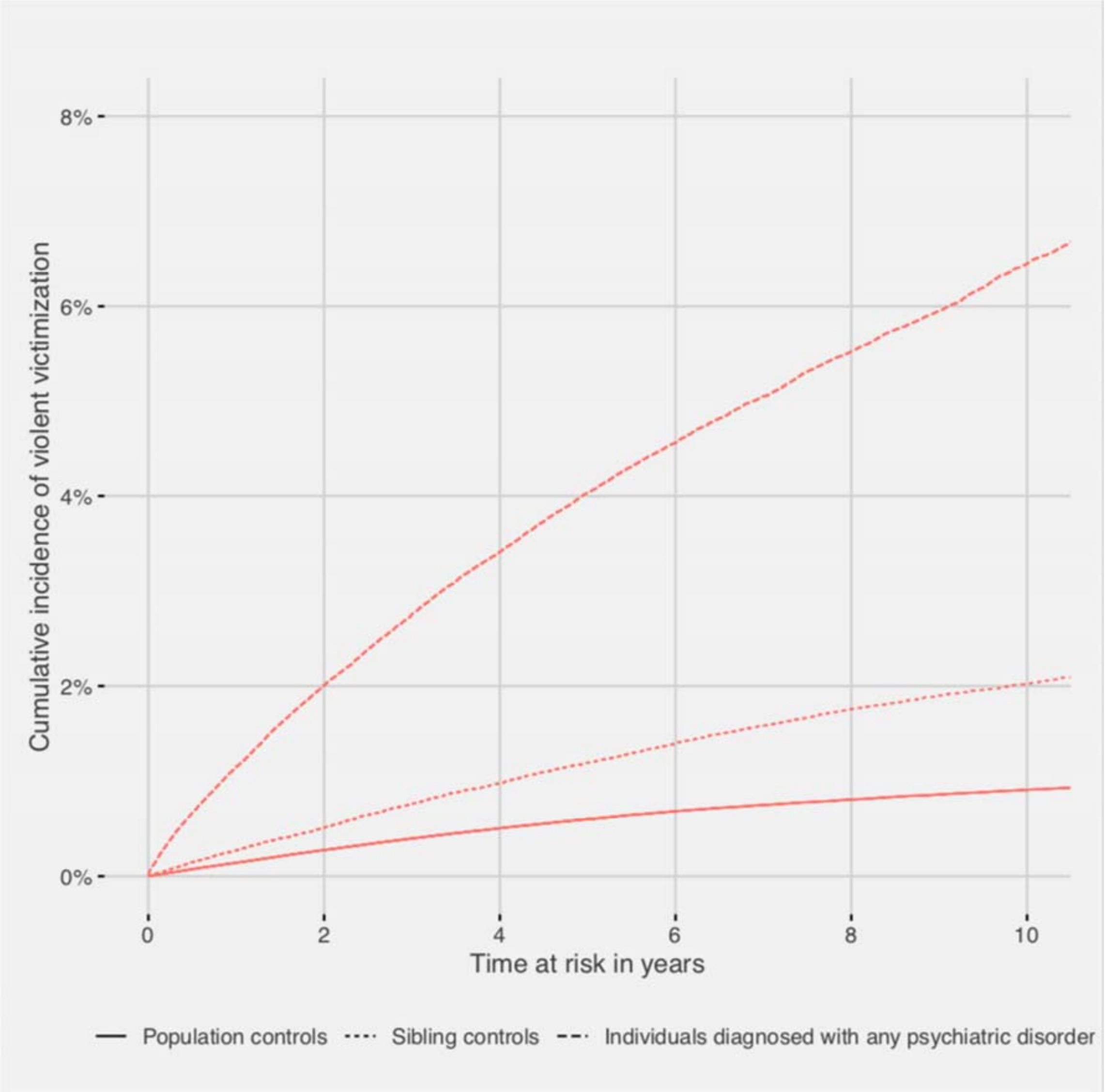

© 2020 Sariaslan A et al. *JAMA Psychiatry*

eFigure 2. Sex and Age-Adjusted Cumulative Incidence Rates of Perpetration of Violence Across the First 10 Years After the Onset of Any Psychiatric Disorder in Patients Compared With Unaffected Siblings and General Population Control Groups

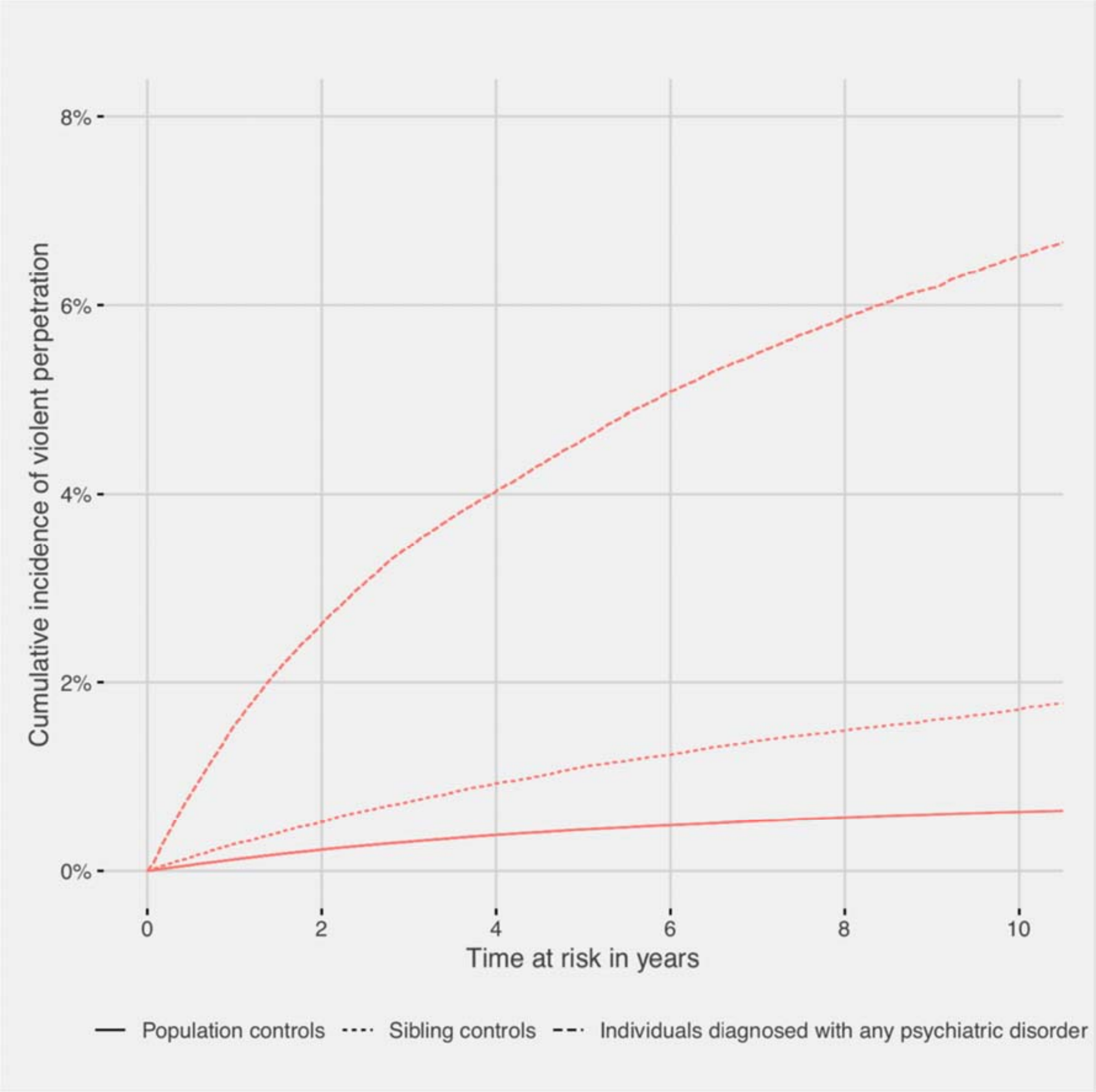

© 2020 Sariaslan A et al. *JAMA Psychiatry*

eFigure 3. Sensitivity Tests: Alternative Exposure and Outcome Definitions and Matching Criteria

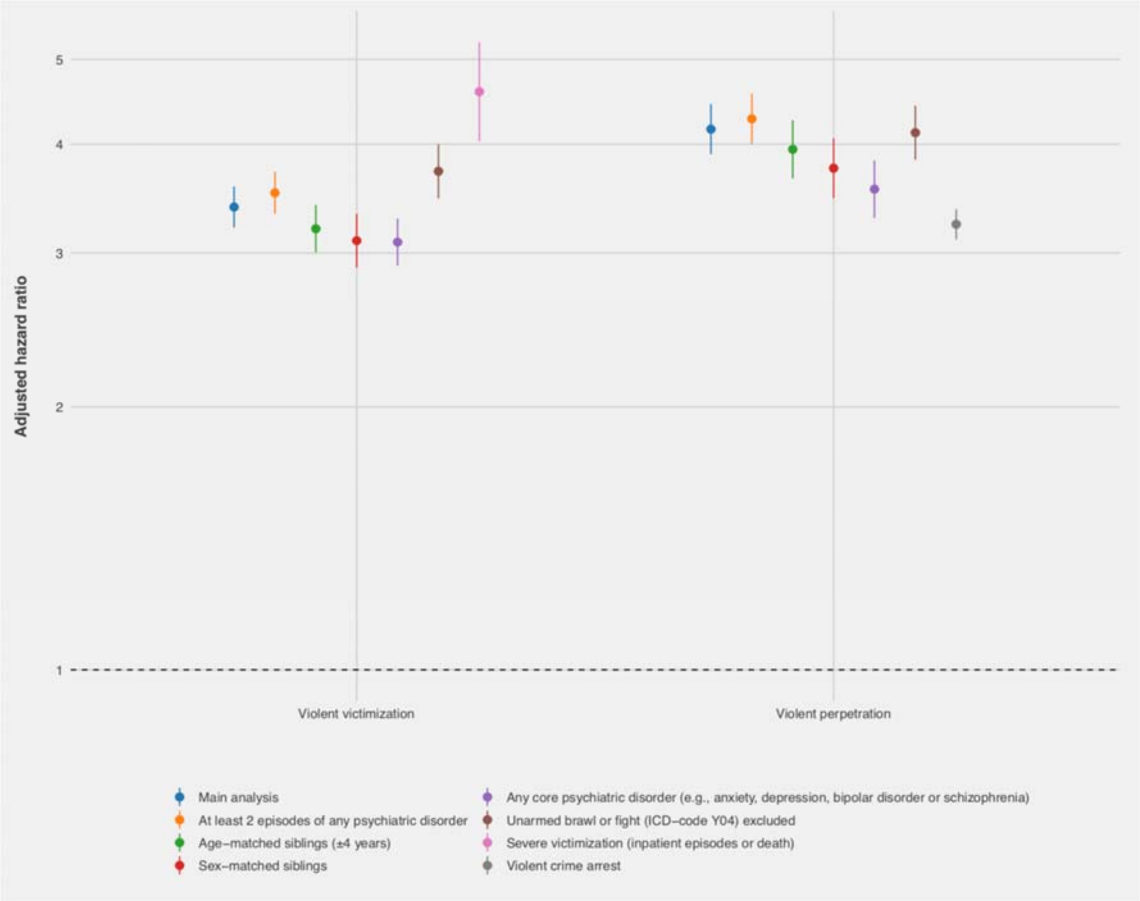

Note: The adjusted hazard ratios refer to within-family estimates comparing differentially exposed siblings and adjusted for sex, birth year, birth order and the individual's history of violent victimization and perpetration. As the comparisons are made within families, there is no need to adjust for factors that are constant within families.

eFigure 4. Sensitivity Test: Associations Between Specific Psychiatric Disorders and Subsequent Risks of Subjection to Violence and Perpetration of Violence With and Without Adjustments for All Other Psychiatric Disorders

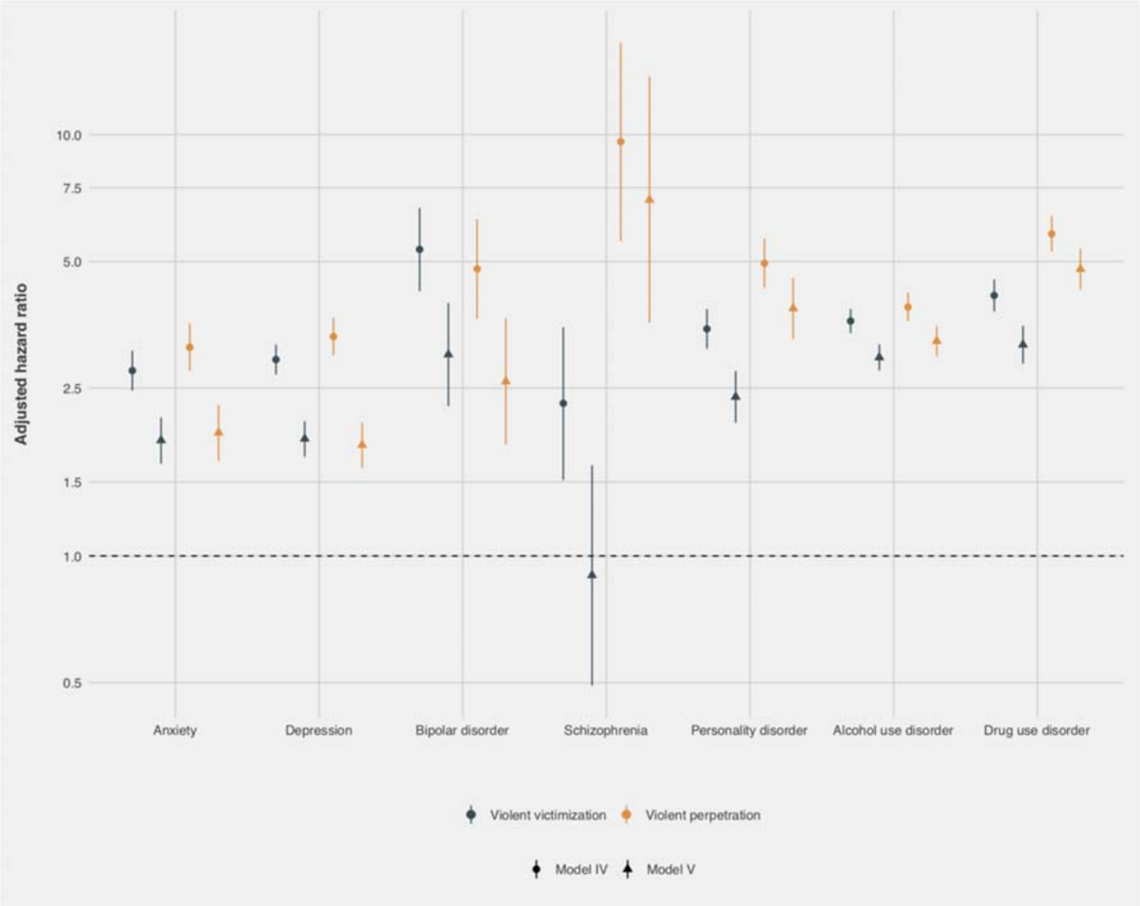

Note: Model IV: Within-family estimates comparing differentially exposed siblings and adjusted for sex, birth year, birth order and the individual's history of violent victimization and perpetration. As the comparisons are made within families, there is no need to adjust for factors that are constant within families. Model V: Model IV + mutually adjusted for all other psychiatric conditions.

© 2020 Sariaslan A et al. *JAMA Psychiatry*

eReferences.

1 Sariaslan A, Långström N, D'Onofrio B, Hallqvist J, Franck J, Lichtenstein P. The impact of neighbourhood deprivation on adolescent violent criminality and substance misuse: A longitudinal, quasi-experimental study of the total Swedish population. *Int J Epidemiol* 2013; **42**: 1057–66.

2 Sariaslan A, Larsson H, D'Onofrio B, Långström N, Lichtenstein P. Childhood family income, adolescent violent criminality and substance misuse: quasi-experimental total population study. *Br J Psychiatry* 2014; **205**: 286–90.

3 Aaltonen M, Kivivuori J, Martikainen P, Sirén R. Socioeconomic differences in violent victimization: Exploring the impact of data source and the inclusivity of the violence concept. *Eur J Criminol* 2012; **9**: 567–83.

4 Aaltonen M, Martikainen P, Moustgaard H, Peltonen R, Remes H. Childhood family income and violent victimization during youth and young adulthood. *J Interpers Violence* 2016; **31**: 2338–59.

5 Johnson KL, Desmarais SL, Van Dorn RA, Grimm KJ. A typology of community violence perpetration and victimization among adults with mental illnesses. *J Interpers Violence* 2015; **30**: 522–40.

6 Ludvigsson J, Andersson E, Ekbom A, *et al.* External review and validation of the Swedish national inpatient register. *BMC Public Health* 2011; **11**: 450.

7 Långström N, Grann M, Tengström A, Lindholm N, Woodhouse A, Kullgren G. Extracting data in file-based forensic psychiatric research: Some methodological considerations. *Nord J Psychiatry* 1999; **53**: 61–7.

8 Brottsförebyggande rådet (The Swedish National Board for Crime Prevention). Det grova våldet i sjukvårdsdata: En metodstudie [The severe violence in hospital data: A methodological study]. Stockholm: The Swedish National Board for Crime Prevention, 2007.

9 Sariaslan A, Lichtenstein P, Larsson H, Fazel S. Triggers for violent criminality in patients with psychotic disorders. *JAMA Psychiatry* 2016; **73**: 796–803.

10 Walter F, Carr M, Mok P, *et al.* Multiple adverse outcomes following first discharge from inpatient psychiatric care: a national cohort study. *The Lancet Psychiatry* 2019; **6**: 582–9.

11 Fazel S, Långström N, Hjern A, Grann M, Lichtenstein P. Schizophrenia, substance abuse, and violent crime. *JAMA* 2009; **301**: 2016.

12 Sellgren C, Landén M, Lichtenstein P, Hultman CM, Långström N. Validity of bipolar disorder hospital discharge diagnoses: file review and multiple register linkage in Sweden. *Acta Psychiatr Scand* 2011; **124**: 447–53.

13 Rück C, Larsson KJ, Lind K, *et al.* Validity and reliability of chronic tic disorder and obsessive-compulsive disorder diagnoses in the Swedish National Patient Register. *BMJ Open* 2015; **5**: e007520–e007520.

14 Fazel S, Wolf A, Chang Z, Larsson H, Goodwin GM, Lichtenstein P. Depression and violence: a Swedish population study. *Lancet Psychiatry* 2015; **2**: 224–32.

© 2020 Sariaslan A et al. *JAMA Psychiatry*
